# Insulin or Dapagliflozin Potentially Improves Microcirculatory Dysfunction or INOCA Related to Culprit Vessels in Diabetic Patients With STEMI After DCB Therapy During Long‐Term Follow‐Up

**DOI:** 10.1111/1753-0407.70197

**Published:** 2026-02-16

**Authors:** Wei You, Xianglian Ma, Yan Xu, Xiaoping Jin, Wenxing Mao, Meili Ji, Tianyi Huang, Peina Meng, Tian Xu, Yifei Wang, Tianlei Zhang, Zhiming Wu, Fei Ye, Xiangqi Wu

**Affiliations:** ^1^ Department of Cardiology Nanjing First Hospital, Nanjing Medical University Nanjing China; ^2^ Department of Cardiology Mingguang People's Hospital Chuzhou China; ^3^ Department of Cardiology Nanjing Tianyinshan Hospital & The Affiliated Hospital of China Pharmaceutical University Nanjing China; ^4^ Department of Geriatric Nanjing First Hospital, Nanjing Medical University Nanjing China

**Keywords:** DCB, diabetes, drug treatment, INOCA, STEMI

## Abstract

**Background:**

This study aims to investigate the relationship between risk factors and IRA‐related microcirculatory dysfunction, as well as its pharmacological intervention in the context of STEMI treatment using DCB over a two‐year period, especially in those with diabetes.

**Methods:**

This retrospective study enrolled 297 consecutive eligible patients diagnosed with STEMI who received DCB treatment from two centers. Clinical and procedure‐related parameters were collected. AMR and adverse cardiac events were recorded.

**Results:**

Immediately after DCB therapy, IRA‐AMR in non‐diabetes or good‐glycemic control group was significantly smaller than that in diabetes or poor‐glycemic control group (*p* < 0.01). Compared with insulin or dapagliflozin group, IRA‐AMR in non‐insulin or non‐dapagliflozin group was significantly higher (*p* < 0.01). Univariate and multivariate Cox regression indicated that diabetes was a significant predictor of IRA‐INOCA after a 2‐year follow‐up (*p* < 0.05). Furthermore, administration of anti‐diabetic medications and poor glycemic control post DCB treatment surfaced as significant predictors (*p* < 0.01). Diabetic patients exhibited a significantly higher incidence of cardiac death along with IRA‐INOCA complications compared to their non‐diabetic counterparts (*p* < 0.01). IRA‐AMR in diabetic individuals immediately following DCB treatment was significantly lower than those in individuals who experienced inadequate‐glycemic management and were readmitted due to IRA‐INOCA complications (*p* < 0.01). The incidence of IRA‐INOCA in good‐glycemic control, insulin, or dapagliflozin group was significantly lower compared with that in poor‐glycemic control, non‐insulin, or non‐dapagliflozin group (*p* < 0.05).

**Conclusions:**

This finding highlights the importance of managing glycemic control, especially using insulin or dapagliflozin, in diabetic patients with STEMI after DCB treatment. Such measures may improve long‐term cardiovascular outcomes.

## Highlights

- In—hospital analysis indicated that diabetes mellitus (DM) was closely associated with infarct—related artery (IRA)—coronary microcirculatory dysfunction (CMD) in ST—segment elevation myocardial infarction (STEMI) patients immediately after the drug—coated balloon (DCB) treatment. Good glycemic control, particularly through the use of insulin or dapagliflozin, could reverse this abnormal status.
- Two—year follow—up results showed that DM was a significant predictor for IRA—ischemia with non—obstructive coronary arteries (INOCA) in STEMI patients after DCB treatment. Poor glycemic control exacerbated CMD in diabetic patients with STEMI after the treatment. Good glycemic control, especially with the use of insulin or dapagliflozin, could significantly reduce the incidence of IRA—INOCA, thereby improving long—term prognosis.

## Graphical abstract

This finding highlights the critical importance of closely managing glycemic control, especially when using insulin or dapagliflozin, in diabetic patients with STEMI after DCB treatment. Such measures may potentially improve microcirculatory dysfunction and reduce the risk of IRA‐INOCA, thereby enhancing long‐term cardiovascular outcomes. BG, blood glucose; MI, myocardial infarction.

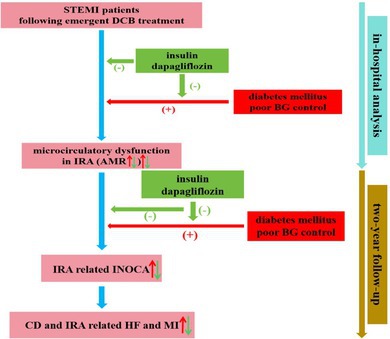

## 1 Introduction

In recent years, drug‐coated balloons (DCB) have increasingly demonstrated their safety and efficacy for emergency revascularization in patients with ST‐segment elevation myocardial infarction (STEMI) [1, 2]. The application of DCB has emerged as a promising therapeutic strategy for managing individuals experiencing STEMI [1, 2]. These devices deliver antiproliferative agents directly to the coronary artery wall, effectively inhibiting neointimal hyperplasia and reducing the risk of in‐stent restenosis (ISR) [3, 4, 5, 6]. This approach is particularly beneficial for diabetic patients, who are often predisposed to heightened neointimal proliferation and recurrent ISR following percutaneous coronary intervention (PCI) [7, 8]. Moreover, DCB offers the advantage of minimizing the prolonged use of dual antiplatelet therapy, which can be especially advantageous for diabetic patients at an increased risk of bleeding complications [3, 4, 5, 6]. However, some patients continue to experience poor long‐term outcomes [3, 4, 5, 6, 9], highlighting the need for further research into the factors influencing the prognosis of these patients and the possible underlying mechanisms involved.

Despite the advantages of DCB, the prognosis of STEMI patients, particularly those with diabetes mellitus (DM), remains suboptimal [10]. The presence of DM not only increases the risk of neointimal hyperplasia but also contributes to microvascular dysfunction, which can lead to ischemia with non‐obstructive coronary arteries (INOCA) [11, 12, 13]. INOCA is a condition characterized by myocardial ischemia in the absence of significant epicardial coronary artery stenosis, and it is increasingly recognized as an important contributor to adverse cardiac events in STEMI patients [11]. Therefore, understanding the relationship between DM and microcirculation is crucial for improving the long‐term outcomes of these patients after emergent DCB treatment. Furthermore, there is no study to date to report that the impact of glycemic control on the incidence of INOCA in STEMI patients treated with DCB, especially in the context of long‐term follow‐up. Prior studies have primarily focused on the immediate effects of DCB on neointimal hyperplasia and restenosis rates, with limited attention to the broader cardiovascular implications, such as microvascular dysfunction and its clinical sequelae.

Considering that diabetic patients face an increased risk of microvascular dysfunction, examining the role of glycemic control in this mechanism could provide significant insights into improving the management of STEMI patients undergoing DCB treatment. The state of residual cardiomyocytes supplied by the infarct‐related artery (IRA) is crucial for the prognosis of STEMI patients [14, 15]. Thus, the current study aims to evaluate the correlation between glycemic control or the administration of anti‐diabetic medications and the occurrence of IRA‐related microvascular dysfunction in diabetic STEMI patients treated with DCB in the hospital. Furthermore, this study also aims to investigate the impact of glycemic control on microvascular dysfunction or IRA‐INOCA and long‐term cardiovascular outcomes in this group. By tackling these issues, we aim to improve our comprehension of the factors that affect the prognosis of diabetic STEMI patients post‐DCB treatment and to pinpoint potential approaches for enhancing their long‐term cardiovascular well‐being.

## 2 Methods

### 2.1 Patients

This study was designed as a retrospective cohort analysis. The research protocol has been approved by the ethics committees of both Nanjing First Hospital, affiliated with Nanjing Medical University, and Mingguang People's Hospital. The research was carried out in compliance with the Declaration of Helsinki. A total of 336 patients diagnosed with STEMI who experienced initial onset within 12 h were effectively treated with DCB at the Division of Cardiology in both Nanjing First Hospital, affiliated with Nanjing Medical University, and Mingguang People's Hospital from January 1, 2019, to August 30, 2023. These patients underwent a follow‐up period of 2 years.

**FIGURE 1:**
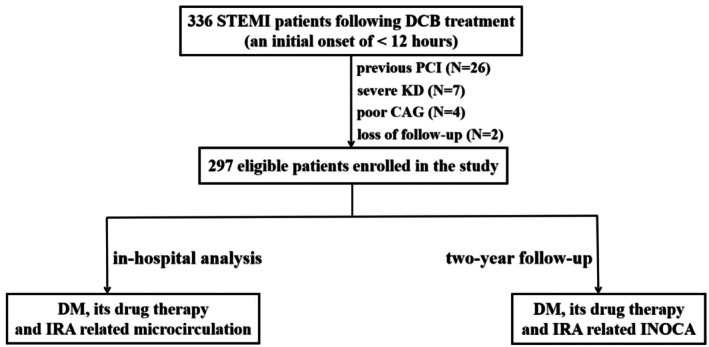
Flow diagram of the study patients. CAG, coronary angiography; DCB, drug‐coated balloon; DM, diabetes mellitus; INOCA, ischemia and non‐obstructive coronary artery disease; IRA, infarct‐related artery; KD, kidney dysfunction; PCI, percutaneous coronary intervention; STEMI, ST‐segment elevation myocardial infarction.

We enrolled a consecutive series of patients from the two medical centers. The inclusion criteria required that participants be diagnosed with STEMI and subsequently receive timely and effective DCB treatment; all patients had de novo coronary artery lesions with stenosis (DS ≥ 70%) suitable for DCB treatment, as confirmed by coronary angiography (CAG); all participants had to be over the age of 18. Exclusion criteria included individuals under the age of 18, those with a history of prior PCI or coronary artery bypass grafting (CABG), patients with left main disease and chronic total occlusions, patients with contraindications to antiplatelet therapy or severe comorbidities, patients diagnosed with coronary spasm disease, myocarditis, Takotsubo syndrome, severe renal dysfunction (eGFR < 30 mL/min/1.73 m^2^), instances where angiographic video quality was deemed poor, and any patients lost to follow‐up during the two‐year study period. Among those screened for eligibility, 26 patients had a history of PCI; seven presented with severe renal dysfunction; four exhibited suboptimal results in their CAG assessments; and two were lost to follow‐up during the course of this study. Ultimately, we enrolled a total of 297 eligible participants in our research. We also conducted an analysis of the relationship between diabetes or pharmacological treatment and IRA—microcirculation or INOCA during hospitalization and in 2‐year follow‐up assessments (Figure 1).

In the present study, the criteria for effective DCB treatment were established as follows: residual coronary stenosis must be less than 50%; the Thrombolysis in Myocardial Infarction (TIMI) flow grade should be 3; and there must be no flow‐limiting dissection classified as Type C or higher [16]. If these patients underwent ineffective DCB treatment, a drug‐eluting stent (DES) was utilized as a remedial measure.

### 2.2 Data Collection

Detailed clinical data were meticulously extracted from the patients' medical records. This encompassed baseline demographic information, including age and gender. Cardiovascular risk factors, such as hypertension, hyperlipidemia, diabetes, and smoking status, were systematically recorded. A comprehensive history of previous medications—covering aspirin, statins, angiotensin‐converting enzyme inhibitors/angiotensin receptor blockers/angiotensin receptor‐neprilysin inhibitors (ACEI/ARB/ARNI), beta‐blockers, calcium channel blockers (CCB), and anti‐diabetes drugs—was also collected. Additionally, laboratory results related to routine blood tests, biochemical indices, and echocardiographic data were collected.

Quantitative coronary angiography (QCA) analyses were performed using an off‐line QCA system (CASS; Pie Medical Instruments, Maastricht, The Netherlands). The reference vessel diameter (RVD), minimum lumen diameter (MLD), and diameter stenosis (DS) following DCB treatment were measured. A DS ≥ 70% was defined as a severe stenosis lesion, and a DS = 100% was defined as a total occlusive lesion.

### 2.3 DCB Protocol, Lesion Characteristics, and Adjunctive Pharmacotherapy

Regarding the DCB (Paclitaxel‐coated balloon) protocol, as previously mentioned, each DCB was utilized to treat the culprit vessel for approximately 60 s. The selection of the DCB size was based on the vessel diameter, ensuring an appropriate fit for optimal treatment. In terms of lesion characteristics, detailed assessments were conducted prior to the DCB procedure. Details about the maximum and minimum diameters of the DCB, the total length of the DCB, and the total number of DCBs were recorded.

The length of the lesion, its location within the culprit vessel, and the degree of stenosis were all carefully evaluated using QCA.

As for adjunctive pharmacotherapy, patients received a combination of medications. Antiplatelet agents, such as aspirin and clopidogrel/Ticagrelor, were routinely prescribed to reduce the risk of thrombosis. Statins were also administered to manage lipid levels and stabilize atherosclerotic plaques. Additionally, beta‐blockers and either angiotensin‐converting enzyme inhibitors, angiotensin receptor blockers, or angiotensin receptor–neprilysin inhibitors were used to improve cardiac remodeling, depending on the patient's individual condition.

### 2.4 Definition

The diagnosis of STEMI is established through a comprehensive evaluation that includes clinical manifestations indicative of myocardial ischemia, electrocardiographic findings consistent with the criteria for STEMI, and elevated levels of high‐sensitivity troponin. This assessment adheres to the guidelines set forth in the Fourth Universal Definition of Myocardial Infarction [17].

Firstly, the diagnosis of DM is established based on either a documented history of DM or a new diagnosis characterized by a GHb level of 6.5% or higher, a fasting blood glucose level of 7.0 mmol/L or greater, a random blood glucose level of 11.1 mmol/L or above, or an oral glucose tolerance test result indicating a glucose level of 11.1 mmol/L or higher [18, 19]. Secondly, individuals undergoing treatment for diabetes, such as taking oral hypoglycemic agents or insulin, are also considered diabetic.

Poor glycemic control is defined as a GHb level exceeding 6.5% or a fasting blood glucose level surpassing 7.0 mmol/L upon repeated measurements during the follow‐up period [18, 19].

The IRA‐INOCA cohort was defined as a condition of patients with acute STEMI who underwent emergency direct DCB angioplasty and were readmitted because of chest discomfort. These patients had CAG indicating stenosis of less than 50% in the culprit vessel. Electrocardiogram (ECG) or radionuclide imaging confirmed ischemia in the myocardial region supplied by the culprit vessel, with other potential causes of myocardial ischemia, such as myocarditis or Takotsubo syndrome, being excluded [11].

Myocardial infarction with nonobstructive coronary arteries (MINOCA), a specific subset of INOCA, is characterized as a clinical condition wherein patients exhibit signs of acute myocardial infarction (AMI) while simultaneously presenting with non‐obstructive coronary arteries. This is characterized by stenosis of less than 50% in any epicardial coronary artery [20, 21]. This definition is consistent with the fourth universal definition of AMI and explicitly excludes myocarditis and Takotsubo syndrome from the final diagnosis of MINOCA [17, 20].

Cardiac death (CD) arises from myocardial infarction, severe arrhythmias, or heart failure [22]. Heart failure was diagnosed following previous guidelines [23]. IRA‐related heart failure is defined as heart failure resulting from an IRA or its microcirculation [23].

### 2.5 Follow‐Up

Clinical follow‐up was routinely conducted at 1, 3, 6, 12, 18, 24 months via office visits or telephone interviews. During this period, data on the occurrence of IRA‐INOCA, along with other major adverse cardiac events (MACEs) such as recurrent MI, target lesion revascularization, and CD, were collected.

Follow‐up events were adjudicated by experienced cardiologists who had no involvement in the study. They examined all available clinical data, including medical records, imaging studies, and laboratory results, to determine whether each event met the predefined criteria for IRA‐INOCA or other MACEs. This process ensured objective and consistent adjudication of events throughout the study. Moreover, the statistical analysts who carried out the data analysis remained blinded until the final analysis was completed.

### 2.6 Calculation of the Angiographic Microvascular Resistance (AMR)

The AMR was generated utilizing the QFR software (AngioPlus Core, version V3, Shanghai Pulse Medical Technology Inc., Shanghai, China) [24]. This advanced software is capable of automatically delineating the lumen contour of the coronary artery and calculating flow velocity by dividing the length of the vascular centerline by the contrast agent filling time. By employing fluid mechanics equations, the software further computes distal coronary artery pressure and subsequently divides this pressure by flow velocity to obtain the AMR.

The acquisition method for AMR is outlined as follows: First, import the collected high‐quality coronary angiography data into the software system. Second, initiate the automatic vascular contour recognition program. Next, manually correct any significant discrepancies between the automated vascular reconstruction and the actual vascular contours. Finally, launch the AMR calculation program, which takes approximately 30 s to complete; thereafter, the AMR can be obtained.

In our study, two independent operators, who were blind to the patients' data and clinical outcomes, conducted a blinded analysis.

### 2.7 Statistical Analysis

Categorical variables were presented as counts with corresponding percentages, while continuous variables were reported as means accompanied by standard deviations (SD) or medians along with interquartile ranges (IQRs). The comparison of categorical variables was conducted using the *χ* ^2^ test. To evaluate the distributions of continuous variables, the Kolmogorov–Smirnov test was utilized. Continuous variables that followed a normal distribution were expressed as mean ± SD, whereas non‐normally distributed data were represented as medians with IQRs. For normally distributed continuous variables, the independent samples *t*‐test was employed for comparisons between two groups. In cases where continuous variables did not follow a normal distribution, the Mann–Whitney *U* test was applied for two‐group comparisons. For missing data, we employed multiple imputation techniques to minimize potential bias. This approach generates several plausible datasets by filling in missing values based on the observed data distribution, allowing for a more robust analysis.

The Kaplan–Meier method was employed to derive event rates during follow‐up and to construct time‐to‐event curves, which were subsequently compared using the log‐rank test. For confounding adjustments, a clear list of potential confounders considered in the study was presented. These confounders were identified based on prior literature and clinical knowledge. To identify predictors of INOCA within the IRA, a univariate Cox regression analysis was performed. Variables identified as significant in this initial analysis were incorporated into a multivariate model. The outputs included hazard ratios (HR), 95% confidence intervals (CI), and *p*‐values. Sensitivity analyses were also conducted to assess the robustness of our findings, such as by excluding patients with missing key data points for handling confounders. All statistical tests conducted were two‐tailed, with statistical significance established at 0.05. Statistical analyses were carried out using SPSS version 20.0 (SPSS Institute Inc.).

## 3 Results

### 3.1 Basic Clinical Data in Patients With STEMI Following DCB Treatment

Upon reviewing the data presented in Table 1, it is clear that a significant majority of patients who experienced STEMI following treatment with DCB were male, accounting for 75.08% of the total patient population. The average age of these individuals was 63.00 years, indicating that this condition predominantly affects middle‐aged and elderly populations. In terms of risk factors, a considerable proportion of patients were current smokers (63.64%) and had hypertension (62.63%), followed by hyperlipidemia (44.11%), and DM (32.32%). Regarding their prior medication history, aspirin and statins emerged as the most commonly utilized drugs, with usage rates recorded at 50.51% and 59.26%, respectively. The onset time of symptoms, recorded as the median with interquartile range, was 7 h, indicating the time from symptom onset to treatment initiation. Laboratory findings revealed an average red blood cell (RBC) count of 4.54 × 10^12^/L, an average white blood cell (WBC) count of 8.59 × 10^9^/L, and an average platelet (PLT) count of 198.31 × 10^9^/L. Concerning lipid profiles, the mean total cholesterol (TC) level was found to be 4.39 mmol/L; low‐density lipoprotein cholesterol (LDL‐C) averaged at 2.69 mmol/L while high‐density lipoprotein cholesterol (HDL‐C) averaged at 0.98 mmol/L.

**TABLE 1:** Basic clinical data in patients with STEMI following DCB treatment.

Variables	Patients (*n* = 297)
Demographics	
Gender (male/female)	223/74
Age (year)	63.00 ± 12.03
Risk factors	
Current smoker (%)	63.64
Hypertension (%)	62.63
Hyperlipemia (%)	44.11
Diabetes mellitus (%)	32.32
Previous drug history	
Aspirin (%)	50.51
Statin (%)	59.26
ACEI/ARB/ARNI (%)	32.66
Beta‐blocker (%)	40.07
CCB (%)	33.33
Anti‐diabetes drug (%)	20.88
Onset Time (h)	7.00 (4.00, 9.00)
Laboratory results	
RBC (×10^12^/L)	4.54 ± 2.43
WBC (×10^9^/L)	8.59 ± 2.73
PLT (×10^9^/L)	198.31 ± 59.71
TC (mmol/L)	4.39 ± 1.17
LDL‐C (mmol/L)	2.69 ± 0.95
HDL‐C (mmol/L)	0.98 ± 0.23
TG (mmol/L)	1.49 (1.07, 2.18)
FBG (mmol/L)	6.25 ± 1.38
GHb (%)	6.05 ± 1.03
BUN (mmol/L)	6.08 ± 4.02
Scr (μmol/L)	79.58 ± 40.14
Serum D‐dimer (μg/mL)	0.35 (0.20, 0.60)
Serum fibrinogen (g/L)	3.43 ± 1.10
hs‐CRP (μg/mL)	6.27 (2.63, 18.40)
NT‐proBNP (pg/mL)	328.00 (152.67, 805.55)
LVEF (%)	56.74 ± 8.08
Anti‐diabetes drug post ePCI (%)	30.98

Abbreviations: ACEI, angiotensin‐converting enzyme inhibitors; ARB, angiotensin receptor blockers; ARNI, angiotensin receptor‐neprilysin inhibitors; BUN, blood urea nitrogen; CCB, Calcium channel blockers; DCB, drug‐coated balloon; ePCI, emergent percutaneous coronary intervention; FBG, fasting blood glucose; GHb, glycosylated hemoglobin; HDL‐C, high‐density lipoprotein cholesterol; hs‐CRP, high‐sensitivity C‐reactive protein; LDL‐C, low‐density lipoprotein cholesterol; LVEF, left ventricular ejection fraction; NT‐proBNP, N‐terminal pro‐brain natriuretic peptide; PLT, platelet; RBC, red blood cell; Scr, serum creatinine; STEMI, ST‐segment elevation myocardial infarction; TC, total cholesterol; TG, triglycerides; WBC, white blood cell.

Fasting blood glucose (FBG) levels were measured, with an average value of 6.25 mmol/L, which can provide information about the patients' glucose metabolism status. Glycated hemoglobin (GHb) levels were also recorded, averaging at 6.05%, offering a long‐term indicator of blood glucose control. Additional laboratory parameters, including triglycerides (TG), blood urea nitrogen (BUN), serum creatinine (Scr), serum D‐dimer levels, serum fibrinogen levels, high‐sensitivity C‐reactive protein (hs‐CRP), N‐terminal pro‐brain natriuretic peptide (NT‐proBNP), and left ventricular ejection fraction (LVEF), also provided valuable insights into the health status of these patients.

### 3.2 CAG Results and DCB Related Data in Patients With STEMI Following DCB Treatment

Upon reviewing the data presented in Table 2, it is clear that among the 297 patients diagnosed with STEMI who received DCB treatment, a significant proportion of the target vessels were identified as the left anterior descending artery (LAD), which constituted 56.57% of cases. This was followed by the right coronary artery (RCA) at 27.61%, and the left circumflex artery (LCX) at 15.82%. The mean lesion length measured was 30.79 ± 13.85 mm. Notably, the data also revealed that nearly half of the patients, precisely 148 out of 297, presented with total occlusion, while the remaining 149 had severe stenosis. In terms of DCB‐specific metrics, the maximum and minimum diameters of the DCBs were recorded as 2.99 ± 0.51 and 2.86 ± 0.49 mm, respectively. The total length of DCBs utilized averaged at 38.07 ± 15.07 mm, with each patient receiving an average of 1.32 ± 0.51 DCBs. Post‐emergent PCI angiographic results revealed an average minimal lumen diameter (MLD) of 2.23 ± 0.43 mm, a distal reference vessel diameter (RVD) of 2.79 ± 0.47 mm, and a percentage diameter stenosis (DS) averaging at 20.25% ± 7.00%.

**TABLE 2:** Angiographic results and DCB related data in patients with STEMI following DCB treatment.

Variables	Patients (*n* = 297)
Target vessel	
LAD (%)	56.57
LCX (%)	15.82
RCA (%)	27.61
Lesion length (mm)	30.79 ± 13.85
Total occlusion/severe stenosis (%)	148/149
DCB related data	
Maximal DCB diameter (mm)	2.99 ± 0.51
Minimal DCB diameter (mm)	2.86 ± 0.49
Total DCB length (mm)	38.07 ± 15.07
Total DCB number	1.32 ± 0.51
Angiographic results post ePCI	
MLD (mm)	2.23 ± 0.43
Distal RVD (mm)	2.79 ± 0.47
DS (%)	20.25 ± 7.00

Abbreviations: DS, diameter stenosis; LAD, left anterior descending artery; LCX, left circumflex artery; MLD, minimal lumen diameter; RCA, right coronary artery; RVD, reference vessel diameter.

### 3.3 DM, Its Pharmacological Treatment, and IRA‐Related Microcirculation in STEMI Patients Following Emergent DCB Therapy During Hospitalization

**FIGURE 2:**
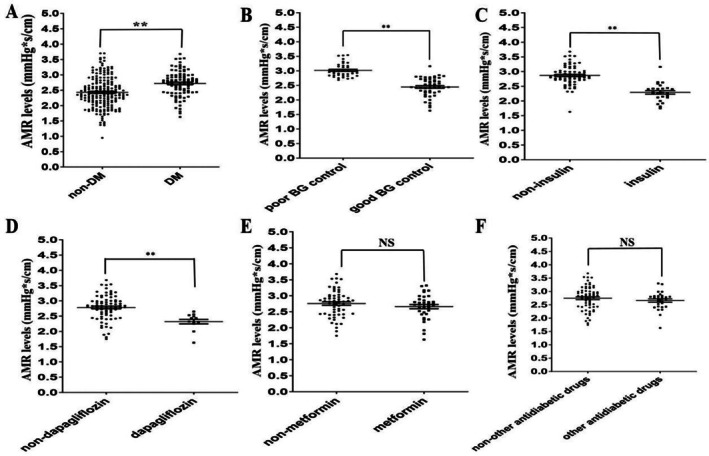
DM, its pharmacological treatment, and IRA‐related microcirculation in STEMI patients following emergent DCB therapy during hospitalization. (A) Comparison between diabetic and non‐diabetic patients. (B) Comparison between the poor BG control group and the good BG control group. (C) Comparison between the non‐insulin use group and the insulin use group. (D) Comparison between the non‐dapagliflozin use group and the dapagliflozin use group. (E) Comparison between the non‐metformin use group and the metformin use group. (F) Comparison between the non‐other diabetic drugs use group and the other diabetic drugs use group. AMR, angiographic microvascular resistance; BG, blood sugar; NS, not significance. **p < 0.01 compared to control groups.

We measure AMR to reflect coronary microcirculation. Immediately after the emergent PCI procedure, the IRA‐related AMR in the non‐DM or good blood glucose (BG) control group was significantly smaller than that in the DM or poor BG control group (*p* < 0.01) (Figure 2A,B). We then evaluated whether any antidiabetic drugs could protect the microcirculation in IRA. Compared with the insulin or dapagliflozin group, the IRA‐related AMR in the non‐insulin or non‐dapagliflozin group was significantly higher (*p* < 0.01) (Figure 2C,D). There were no significant differences in IRA‐related AMR between the metformin or other antidiabetic drugs group (glucosidase inhibitor, sulfonylureas, or thiazolidinedione) and the non‐metformin or non‐other antidiabetic drugs group (*p* > 0.05) (Figure 2E,F).

Totally, these findings suggest that in STEMI patients undergoing emergent DCB therapy, the presence of DM or poorly‐controlled blood glucose levels is associated with impaired IRA‐related microcirculation, as evidenced by higher AMR values. Conversely, the use of insulin or dapagliflozin appears to have a protective effect on microcirculation, reducing AMR compared to non‐use of these drugs. However, metformin and other antidiabetic drugs did not show a significant impact on IRA‐related microcirculation in this study. This information is crucial for clinicians to consider when managing STEMI patients with DM, as optimizing blood glucose control and selecting appropriate antidiabetic medications may potentially improve microcirculation and thus the overall prognosis of these patients.

### 3.4 Clinical Outcomes in Patients With STEMI Following DCB Treatment During 2‐Year Follow‐Up

Upon reviewing the data presented in Table 3, several key clinical outcomes emerge for patients with STEMI who underwent DCB treatment during the two‐year follow‐up period. The incidence of CD is relatively low at seven cases, suggesting a degree of effectiveness of DCB treatment in mitigating fatal cardiac events.

**TABLE 3:** Clinical outcomes in patients with STEMI following DCB treatment during 2‐year follow‐up.

Variables	Patients (*n* = 297)
CD	7
Readmission for ICAD	37
Non‐IRA‐related ICAD	5
IRA‐related INOCA	20
IRA‐related ICAD (TVR)	12
Readmission for MI	15
Non‐IRA related MI	1
IRA‐related MINOCA	6
IRA‐related MI (TVR)	8
Readmission for IHF	17
Non‐IRA related IHF	1
IRA‐related IHF (non‐TVR)	10
IRA‐related IHF (TVR)	6

Abbreviations: CD, cardiac death; ICAD, ischemic coronary artery disease; IHF, ischemic heart failure; MI, myocardial infarction; TVR, target vessel revascularization.

Notably, there are also significant readmission rates due to various causes. A total of 37 readmissions were recorded for ischemic coronary artery disease (ICAD), encompassing different subtypes. Non‐IRA related ICAD accounts for five cases, while IRA‐related ICAD includes both IRA‐related INOCA and IRA‐related ICAD (TVR), with 20 and 12 cases, respectively. Readmissions due to MI further illustrate patterns associated with ICAD. There is one case of non‐IRA related MI; among IRA‐related MI instances, six are classified as IRA‐related MINOCA and eight as IRA‐related MI (TVR). These figures contribute to our understanding of MI recurrence. In terms of ischemic heart failure (IHF), another manifestation linked to ICAD, there are a total of 17 readmissions documented. Non‐IRA related IHF is infrequent, represented by only one case; conversely, IRA‐related IHF comprises 10 non‐TVR cases and six TVR cases. Overall, these clinical outcome data provide essential insights into assessing both the long‐term efficacy and safety profile of DCB treatment in patients diagnosed with STEMI.

### 3.5 Predictors of IRA‐Related INOCA Assessed Through the Cox Regression Model in Patients With STEMI Following DCB Treatment During a Two‐Year Follow‐Up Period

Based on the data presented in Table 4, several key predictors of IRA‐related INOCA in patients with STEMI who underwent DCB treatment during a two‐year follow‐up period can be identified.

**TABLE 4:** Predictors of IRA‐related INOCA using the Cox regression model in patients with STEMI following DCB treatment during a 2‐year follow‐up period.

Variables	Univariate analysis HR (95% CI)	*p*	Multivariate analysis HR (95% CI)	*p*
Gender (male/female)	0.485 (0.198–1.187)	0.113		
Age (year)	1.029 (0.989–1.069)	0.158		
Current smoker (%)	5.378 (1.248–23.180)	0.024	5.339 (1.210–23.561)	0.027
Hypertension (%)	0.953 (0.390–2.332)	0.916		
Hyperlipemia (%)	0.855 (0.350–2.092)	0.732		
Diabetes mellitus (%)	4.120 (1.643–10.326)	0.003	3.438 (1.334–8.864)	0.011
Aspirin (%)	1.547 (0.632–3.784)	0.339		
Statin (%)	1373.764 (0.000–1.121E^111^)	0.955		
ACEI/ARB/ARNI (%)	1.958 (0.654–5.855)	0.230		
Beta‐blocker (%)	0.816 (0.338–1.969)	0.651		
CCB (%)	0.481 (0.200–1.155)	0.101		
Anti‐diabetes drug before admission (%)	0.617 (0.237–1.606)	0.323		
Onset Time (h)	1.132 (0.968–1.324)	0.121		
RBC (×10^12^/L)	1.012 (0.877–1.167)	0.875		
WBC (×10^9^/L)	1.011 (0.862–1.186)	0.891		
PLT (×10^9^/L)	0.996 (0.988–1.004)	0.287		
TC (mmol/L)	1.171 (0.828–1.655)	0.373		
LDL‐C (mmol/L)	1.122 (0.731–1.721)	0.599		
HDL‐C (mmol/L)	0.516 (0.067–3.957)	0.525		
TG (mmol/L)	1.007 (0.710–1.428)	0.968		
FBG (mmol/L)	1.504 (1.262–1.793)	0.000		
GHb (%)	1.668 (1.281–2.172)	0.000		
BUN (mmol/L)	0.912 (0.709–1.174)	0.476		
Scr (μmol/L)	0.994 (0.974–1.014)	0.552		
Serum D‐dimer (μg/mL)	0.211 (0.033–1.360)	0.102		
Serum fibrinogen (g/L)	0.926 (0.612–1.400)	0.715		
hs‐CRP (μg/mL)	0.997 (0.973–1.022)	0.823		
NT‐proBNP (pg/mL)	1.000 (1.000–1.001)	0.011	1.000 (1.000–1.000)	0.819
LVEF (%)	0.949 (0.911–0.990)	0.015	0.971 (0.940–1.002)	0.068
Lesion length (mm)	1.020 (0.990–1.049)	0.191		
Total occlusion (%)	6.009 (1.761–20.505)	0.004	5.195 (1.507–17.904)	0.009
Maximal DCB diameter (mm)	0.883 (0.375–2.080)	0.776		
Minimal DCB diameter (mm)	0.593 (0.234–1.501)	0.270		
Total DCB length (mm)	1.013 (0.985–1.041)	0.370		
DCB number	1.576 (0.738–3.368)	0.240		
MLD post PCI (mm)	0.540 (0.186–1.564)	0.256		
Distal RVD post PCI (mm)	0.795 (0.309–2.047)	0.634		
DS post PCI (%)	1.050 (0.989–1.114)	0.111		
Anti‐diabetes drug post PCI (%)	0.286 (0.117–0.699)	0.006		
Poor blood sugar post PCI (%)	13.076 (5.433–31.468)	0.000		

Abbreviations: HR, hazard ratio; INOCA, ischemia with non‐obstructive coronary arteries; IRA, infarct‐related artery; MINOCA, myocardial infarction with nonobstructive coronary arteries.

Current smoking status emerged as a significant predictor. Patients who were identified as current smokers exhibited a markedly elevated risk, with HR of 5.378 (1.248–23.180) in the univariate analysis and 5.339 (1.210–23.561) in the multivariate analysis, accompanied by corresponding *p*‐values of 0.024 and 0.027, which indicate statistical significance. This finding underscores the importance of implementing smoking cessation programs to mitigate the risk of IRA‐related INOCA within this patient population.

DM emerged as a significant predictor in this context. Patients with DM exhibited an elevated risk, as indicated by HR of 4.120 (1.643–10.326) in the univariate analysis and 3.438 (1.334–8.864) in the multivariate analysis, along with corresponding P values of 0.003 and 0.011, respectively. Another notable predictor was the FBG and GHb level. Patients with higher FBG levels had an increased risk, as reflected by an HR of 1.504 (1.262–1.793) in the univariate analysis with a *P* value of 0.000, indicating strong statistical significance. Similarly, elevated GHb levels were also associated with a higher risk, with an HR of 1.668 (1.281–2.172) in the univariate analysis and a *p*‐value of 0.000. These results highlight the necessity of closely monitoring and controlling blood glucose levels in patients with STEMI receiving DCB treatment to prevent IRA‐related INOCA.

NT‐proBNP levels at admission demonstrated significant predictive value. Elevated NT‐proBNP was correlated with an increased risk of IRA‐related INOCA, exhibiting HR of 1.000 (1.000–1.001) in the univariate analysis and *P*‐values of 0.011. LVEF emerged as another critical predictor; lower LVEF values were linked to a heightened risk, with HR values of 0.949 (0.911–0.990) in the univariate analysis and *p*‐values of 0.015. This highlights the necessity for monitoring and optimizing LVEF in patients following DCB treatment.

Total occlusion was found to be a significant factor. Patients with this condition had an HR of 6.009 (1.761–20.505) in the univariate analysis and 5.195 (1.507–17.904) in the multivariate analysis, both with *p*‐values of 0.004 and 0.009, respectively. This implies that addressing total occlusion is vital in preventing IRA‐related INOCA.

Moreover, following emergent DCB treatment, the long‐term administration of anti‐diabetic medications significantly influenced the incidence of IRA‐related INOCA in patients with STEMI, yielding a HR of 0.286 (0.117–0.699) in the univariate analysis and a *p*‐value of 0.006. This finding suggests that post‐PCI anti‐diabetic therapy may play a critical role in improving the long‐term prognosis for STEMI patients treated with DCB. Additionally, poor glycemic control after PCI emerged as a highly significant predictor, substantially elevating the risk of IRA‐related INOCA (HR = 13.076 [5.433–31.468], *p* = 0.000) in the univariate analysis. This highlights the necessity for rigorous glycemic management within this patient population to mitigate the occurrence of IRA‐related INOCA.

In contrast, several other factors—including age, hypertension, hyperlipidemia, aspirin use, statin use, ACEI/ARB/ARNI use, beta‐blocker use, CCB use, anti‐diabetes drug before admission, onset time, and various blood test results (such as RBC, WBC, PLT, TC, LDL‐C, HDL‐C, TG, BUN, Scr, serum D‐dimer, serum fibrinogen, and hs‐CRP) did not demonstrate significant associations with the risk of IRA‐related INOCA within this cohort. Similarly, lesion length, maximal and minimal DCB diameter, total DCB length, number of DCBs, MLD, distal RVD, and degree of DS did not emerge as significant predictors (*p* > 0.05).

In conclusion, the statistical analysis has yielded valuable insights into the predictors of IRA‐related INOCA in patients with STEMI following DCB treatment. Current smoking status, DM, FBG, GHb, and NT‐proBNP levels at admission, LVEF, and total occlusion have been identified as significant predictors. The long‐term utilization of anti‐diabetic medications post‐DCB treatment positively influences the reduction of IRA‐related INOCA incidence; however, inadequate blood sugar control following PCI significantly elevates risk. These findings underscore the necessity for comprehensive risk assessment and tailored management strategies—including smoking cessation interventions, intensive glycemic monitoring and control, careful monitoring of NT‐proBNP levels and LVEF, as well as timely and effective management of total occlusion to minimize the risk of IRA‐related INOCA in patients with STEMI undergoing DCB treatment.

### 3.6 Comparison of IRA‐Related INOCA and Its Patterns, and CD Between Diabetic and Non‐Diabetic Patients Following DCB Treatment for STEMI

Upon reviewing the data presented in Table 5, it is evident that diabetic patients exhibit a significantly higher incidence of IRA‐INOCA compared to their non‐diabetic counterparts, with rates of 13.54% and 3.48%, respectively. This difference is statistically significant (*p* = 0.000). Similarly, the readmission rates for IRA‐IHF (non‐TVR) and IRA‐MINOCA are markedly elevated in the diabetic group, recorded at 7.29% and 4.17%, respectively, versus 1.49% and 1.00% in the non‐diabetic cohort; both differences achieve statistical significance (*p* = 0.005 and *p* = 0.047). Furthermore, the occurrence of CD is more prevalent among diabetic patients, with a rate of 5.21% compared to just 1.00% in non‐diabetic individuals; this disparity is also statistically significant (*p* = 0.017). These findings underscore the increased risk and poorer prognosis associated with diabetes in patients undergoing DCB treatment for STEMI.

**TABLE 5:** Comparison of IRA‐related INOCA and its patterns, and CD between diabetic and non‐Diabetic patients following DCB Treatment for STEMI.

Variables	DM (*n* = 96)	Non‐DM (*n* = 201)	*p*
IRA‐INOCA (%)	13 (13.54%)	7 (3.48%)	0.000
Readmission for IRA‐IHF (non‐TVR) (%)	7 (7.29%)	3 (1.49%)	0.005
Readmission for IRA‐MINOCA (%)	4 (4.17%)	2 (1.00%)	0.047
CD (%)	5 (5.21%)	2 (1.00%)	0.017

Abbreviation: DM, diabetes mellitus.

**FIGURE 3:**
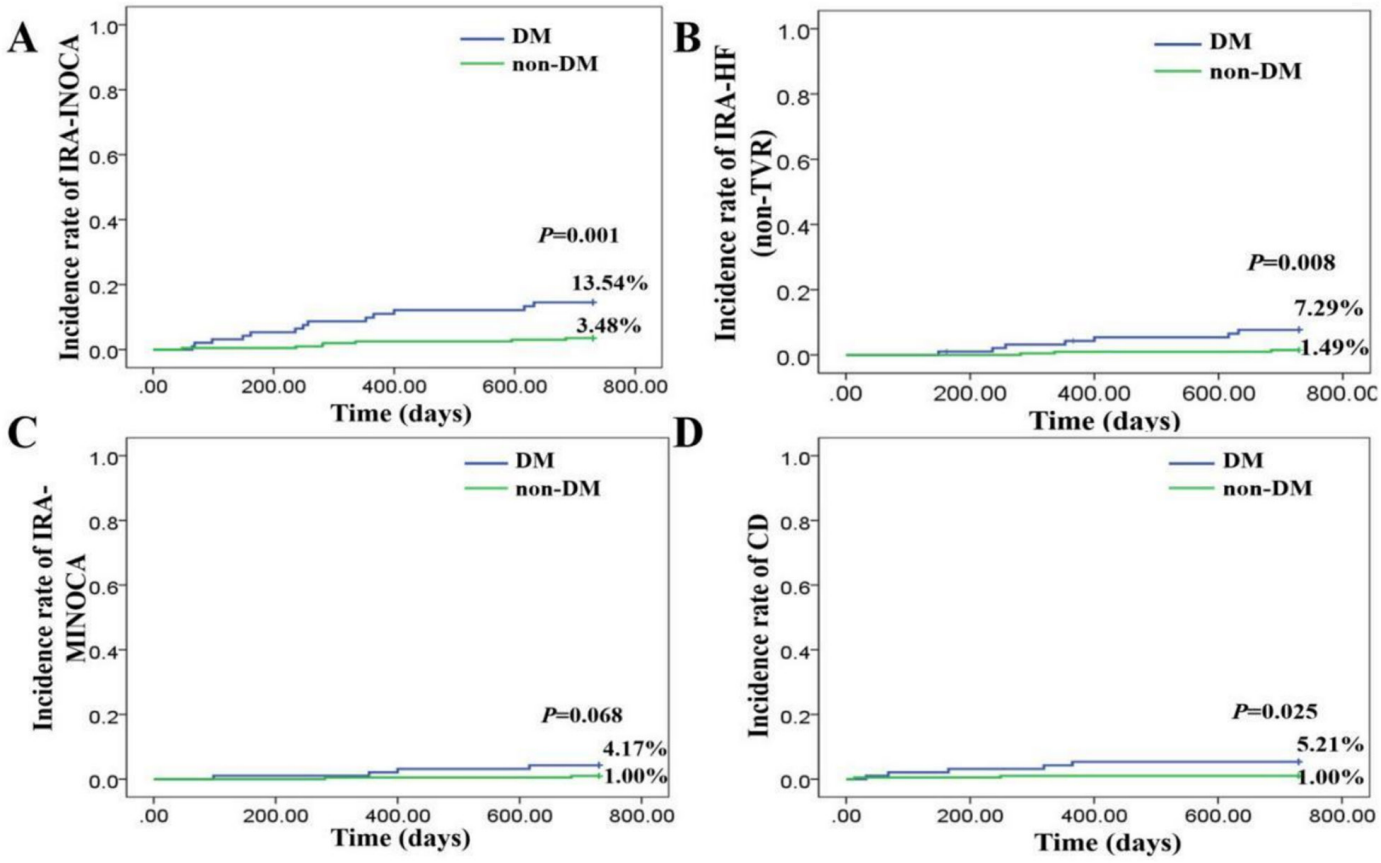
Comparison of IRA‐related INOCA and its patterns, and CD between diabetic and non‐diabetic patients following DCB Treatment for STEMI by the Kaplan–Meier method. CD, cardiac death; HF, heart failure; IRA, infarct‐related artery; MINOCA, myocardial infarction with nonobstructive coronary arteries; TVR, target vessel revascularization.

We also employed the Kaplan–Meier method for comparative analysis (Figure 3). Our results indicated that cumulative event rates for IRA‐INOCA, readmission due to IRA‐IHF (non‐TVR), and CD were significantly lower in diabetic patients than their non‐diabetic counterparts, with *p*‐values of 0.001, 0.008, and 0.025, respectively. Although there was no statistically significant difference observed in cumulative event rates for readmission between diabetic and non‐diabetic patients (*p*‐value = 0.068), the outcomes from the Kaplan–Meier analysis further substantiate our conclusions drawn from Table 5 data. This highlights the necessity for more intensive monitoring and management of diabetic patients with STEMI undergoing DCB treatment.

### 3.7 Comparison of IRA‐Related AMR and INOCA in Patients With STEMI Following Emergent DCB Treatment After Two‐Year Follow‐Up

**FIGURE 4:**
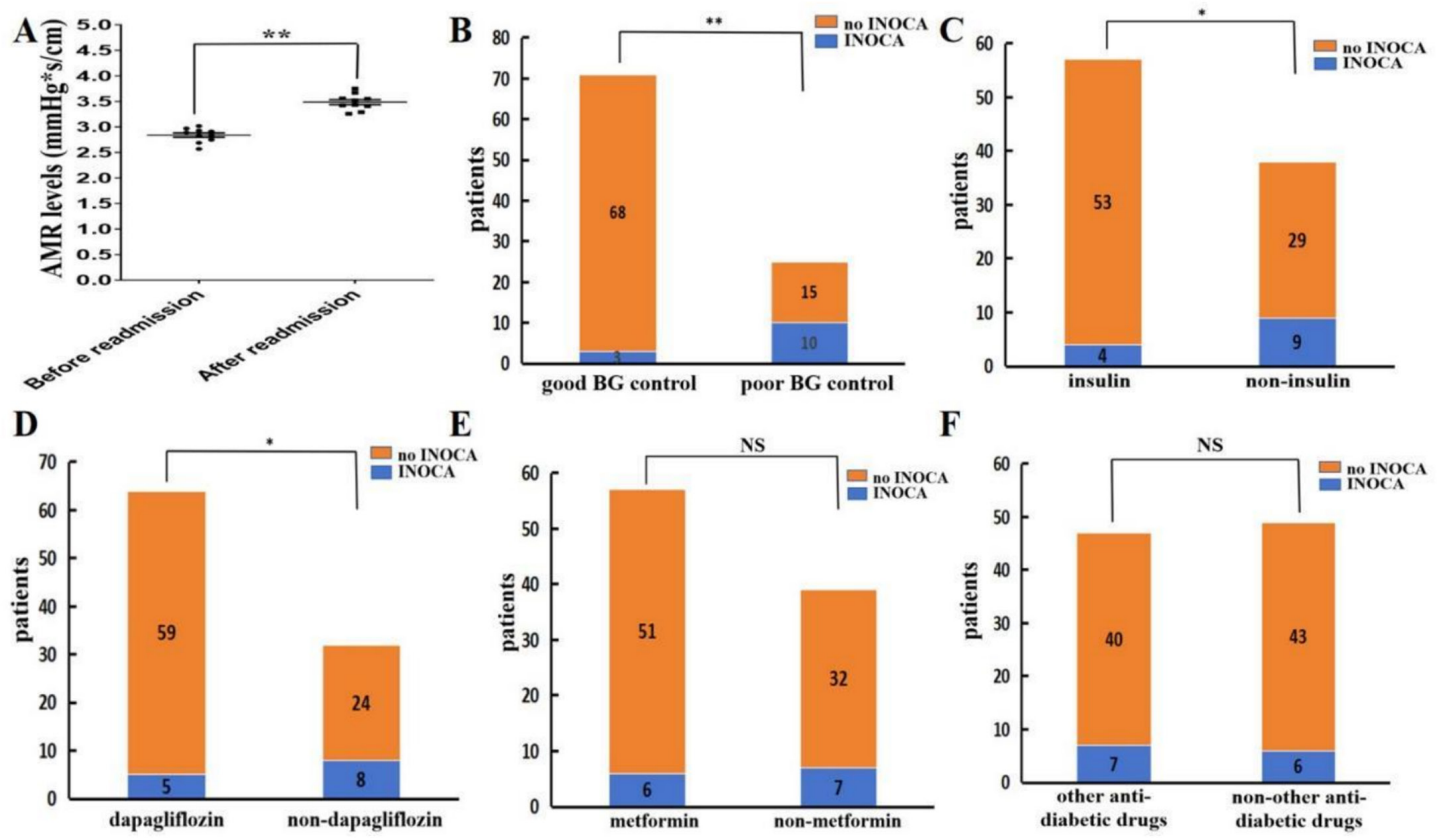
Comparison of IRA‐related AMR and INOCA in patients with STEMI following emergent DCB treatment after two‐year follow‐up. (A) Assessment of IRA‐related AMR levels before and after readmission. (B) Incidence of IRA‐INOCA between the good BG control group and the poor BG control group. (C) Incidence of IRA‐INOCA between the insulin use group and the non‐insulin use group. (D) Incidence of IRA‐INOCA between the dapagliflozin use group and the non‐dapagliflozin use group. (E) Incidence of IRA‐INOCA between the metformin use group and the non‐metformin use group. (F) Incidence of IRA‐INOCA between the other diabetic drugs use group and the non‐other diabetic drugs use group. *p < 0.05, **p < 0.01 compared to control groups.

Thirteen patients with DM were readmitted due to IRA‐related INOCA. Notably, 10 of these patients were identified as having poor blood sugar control. The data indicate that the IRA‐related AMR levels in these individuals immediately following DCB treatment, while experiencing inadequate glycemic management, were significantly lower compared to those who were readmitted solely due to IRA‐related INOCA (*p* < 0.01). This finding implies that suboptimal blood sugar control may exacerbate IRA‐related microcirculatory dysfunction in diabetic STEMI patients who have undergone DCB treatment during long‐term follow‐up (Figure 4A).

We also analyzed IRA‐related INOCA distribution among different populations. The incidence of IRA‐related INOCA in the good BG control, insulin or dapagliflozin group was significantly lower compared with that in the poor BG control, non‐insulin or dapagliflozin group (*p* < 0.01 or *p* < 0.05; Figure 4B–D). There was no significant difference in IRA‐related INOCA between the metformin or other antidiabetic drugs groups and the non‐metformin or non‐other antidiabetic drugs groups (*p* > 0.05; Figure 4E,F).

In conclusion, our study highlights the importance of optimal blood sugar control in diabetic STEMI patients undergoing DCB treatment. Poor glycemic management appears to be closely associated with an increased risk of IRA‐related INOCA and exacerbated microcirculatory dysfunction in the long term. On the other hand, effective blood sugar control strategies, such as the use of insulin or dapagliflozin, can significantly reduce the incidence of IRA‐related INOCA. However, metformin or other antidiabetic drugs do not seem to have a significant impact on preventing IRA‐related INOCA in this patient population. These findings suggest that individualized blood sugar control approaches should be considered for diabetic STEMI patients to improve their long‐term prognosis after DCB treatment.

## 4 Discussion

In the present study, the primary findings are as follows: Firstly, DM was identified as a significant contributing factor to IRA‐related coronary microcirculatory dysfunction (CMD) in patients with STEMI undergoing emergent DCB treatment during the two‐year follow‐up. This dysfunction subsequently heightened the risk of CD, and HF and MI related to the IRA, which represent severe cardiovascular consequences during long‐term follow‐up. Secondly, the study emphasized that inadequate glycemic control exacerbates disturbances in coronary microcirculation related to IRA‐INOCA. Thirdly, well‐controlled blood sugar levels, especially the application of insulin and dapagliflozin, had potential protective effects on CMD associated with IRA‐INOCA in these populations. These findings highlight the necessity for clinicians to closely monitor and manage both DM and CMD health in STEMI patients treated with DCB. In particular, the use of individualized blood sugar control approaches, such as insulin and dapagliflozin, is crucial to mitigate the risk of adverse cardiovascular events after long‐term follow‐up.

Emergent PCI is recognized as the most effective reperfusion therapy for acute STEMI [25]. The implantation of DES during surgical procedures has become standard practice [26]. However, complications such as bleeding associated with dual antiplatelet therapy, ISR, stent thrombosis, and neointimal atherosclerosis continue to present significant challenges [27, 28, 29]. DCBs, which do not induce the chronic inflammation typically associated with metal stents or polymers, have demonstrated advantages in promoting early vascular healing and maintaining normal function [30, 31]. Furthermore, DCBs reduce the risks of ISR and ST, potentially shortening the duration of postoperative dual antiplatelet therapy [30, 31]. Recent studies affirm that emergency DCB treatment is both safe and effective for patients experiencing STEMI, provided they have undergone adequate lesion preparation [1, 2, 16]. In our retrospective study, after a 2‐year follow‐up period, adverse cardiac events—which include CD, recurrent angina, acute MI, target vessel revascularization, and HF—occurred in 14.81% of STEMI patients treated with DCBs. This rate is comparable to that reported in studies involving DES, suggesting that DCB treatment represents a viable strategy for STEMI patients who have received appropriate lesion preparation.

Patients who experience ischemic chest discomfort accompanied by objective signs of myocardial ischemia, yet without obstructive coronary stenosis evident on CAG or coronary computed tomography angiography (CCTA), are classified as having INOCA [11, 32]. CMD is characterized by structural or functional abnormalities in the coronary microvessels, which have diameters less than 500 μm [33]. This microvascular dysfunction plays a crucial role in the pathogenesis of INOCA, leading to symptoms that resemble those observed in obstructive coronary artery disease, such as chest pain and dyspnea [34]. Furthermore, CMD is not only associated with INOCA but also correlates with an increased risk of future cardiovascular events, including CD, MI, and HF [35]. Currently, the index of microcirculation resistance (IMR) is widely employed in clinical settings as a measure of coronary microvascular function [36]. This metric provides a reliable assessment for various clinical conditions such as acute MI, ischemic cardiomyopathy, and microembolism related to interventional therapies that may compromise coronary microcirculation [37]. However, practical challenges—including complex procedures, additional guidewire requirements, and high costs—restrict the widespread application of IMR in clinical practice [36, 37]. In contrast, the index derived from angiographic measurements known as AMR can be obtained swiftly and effectively [24, 38]. Its accuracy and consistency with IMR have been thoroughly validated; therefore, we utilize AMR to evaluate CMD in our study.

In the context of our study, based on in‐hospital analysis, patients with DM who underwent emergent DCB treatment for STEMI had worse CMD, as indicated by the assessment of the AMR. Moreover, during the 2‐year follow‐up, we found that these populations were more likely to have IRA‐INOCA and CMD under poor glycemic control and had a poor prognosis. This finding is consistent with prior research that identifies that DM patients diagnosed with severe lesions suitable for DCB are more prone to have CMD during the 1‐year follow‐up [39]. However, compared to the previous study, our study further delineates the specific subgroup of DM patients with STEMI who are at a heightened risk, namely those with poor glycemic control. This subgroup not only has a propensity to develop CMD but also faces an increased likelihood of adverse cardiovascular outcomes, including IRA‐INOCA. The possible mechanisms underlying this association include endothelial dysfunction, inflammation, and factors induced by oxidative stress, which are further exacerbated by hyperglycemia and insulin resistance [40]. Consequently, diabetic patients experiencing STEMI may be more susceptible to disruptions in IRA‐related coronary microcirculation, leading to an increased risk of IRA‐related INOCA after emergent DCB treatment. This finding in our study has not been previously reported in this context.

Further, these patients exhibited a higher incidence of adverse cardiac events—such as CD, as well as IRA‐related MINOCA and HF in comparison to their non‐diabetic counterparts. This underscores the significant impact of diabetic status on the prognosis of STEMI patients treated with DCB, particularly concerning microcirculatory health and subsequent cardiovascular outcomes. Additionally, initial anti‐diabetic therapies may mitigate the risk of developing INOCA; conversely, inadequate blood glucose control can exacerbate this risk. This highlights the critical need for rigorous glycemic management in this patient population.

Moreover, our study sheds light on the differential impact of various antidiabetic medications on IRA‐CMD and INOCA in diabetic STEMI patients post‐DCB treatment during the 2‐year follow‐up. While insulin and dapagliflozin demonstrated promising protective effects, metformin and other antidiabetic drugs (glucosidase inhibitor, sulfonylureas, or thiazolidinedione) did not exhibit a statistically significant reduction in the IRA‐AMR and the incidence of IRA‐INOCA. This discrepancy may be attributed to the distinct mechanisms of action of these medications on glucose metabolism and vascular function. Insulin, as a direct regulator of blood glucose, may have a more immediate and profound effect on improving microcirculatory function [41, 42]. In contrast, dapagliflozin, through its action on sodium‐glucose co‐transporters, may also contribute to microcirculation protection beyond glycemic control [43, 44]. On the other hand, metformin or other antidiabetic drugs, although effective in lowering blood glucose levels, may not have the same direct impact on coronary microcirculation as insulin or dapagliflozin. Therefore, the choice of antidiabetic medication in diabetic STEMI patients undergoing DCB treatment should be carefully considered, taking into account not only glycemic control but also potential cardiovascular benefits.

Lastly, the presence of current smoking status, elevated NT‐proBNP levels, reduced LVEF, and coronary total occlusion significantly heightens the risk of IRA‐INOCA in patients with STEMI following DCB treatment during long‐term follow‐up. Our findings emphasize the necessity for a comprehensive risk assessment in STEMI patients undergoing DCB therapy, particularly focusing on smoking history, NT‐proBNP levels, LVEF and coronary artery status. For instance, smoking cessation programs and pharmacological interventions to lower NT‐proBNP levels could potentially reduce the risk of IRA‐INOCA in this population. Additionally, optimizing LVEF through guideline‐directed medical therapy and revascularization strategies may also play a pivotal role in mitigating this risk [45, 46]. These measures, when implemented effectively, could contribute to improved cardiovascular outcomes in STEMI patients post‐DCB treatment.

## 5 Conclusions

In conclusion, patients with STEMI who received emergency treatment with DCB to restore patency in the culprit vessel were monitored over a 2‐year period. Our in‐hospital findings indicate that diabetes or inadequate glycemic control may significantly exacerbate microcirculatory disorders associated with the IRA within the heart, primarily evidenced by an increase in AMR. This abnormality could be corrected by insulin or dapagliflozin treatment. During the 2‐year follow‐up, we also found that diabetes or poor glycemic control may exacerbate microcirculatory dysfunction, leading to IRA‐INOCA, as well as subsequent occurrences of sudden cardiac death, heart failure, and myocardial infarction related to this vessel. Treatment with insulin or dapagliflozin could significantly reduce the incidence of IRA‐INOCA.

Moreover, the study highlights the importance of strict glycemic management in patients with STEMI after DCB therapy, especially those with diabetes, to prevent adverse cardiovascular events. The results also suggest that intervention with insulin or dapagliflozin not only improves microcirculatory function but also reduces the risk of long‐term complications such as sudden cardiac death, heart failure, and recurrent myocardial infarction. These findings provide valuable insights for clinicians in optimizing treatment strategies for STEMI patients with diabetes, emphasizing the need for personalized approaches to improve outcomes.

## 6 Limitations

Our study, while providing valuable insights, is not without its limitations. Firstly, the sample size of our study population was relatively small, which may limit the generalizability of our findings to broader patient groups. Larger multicenter studies are essential to confirm the observed associations and to explore potential variations across different demographics and clinical settings.

Secondly, our study employed AMR as a surrogate marker for CMD. Although AMR has shown a strong correlation with IMR and offers practical advantages, it may not fully capture the complexity of coronary microcirculatory dysfunction. Other non‐invasive methods, such as cardiac magnetic resonance imaging (CMR) or positron emission tomography (PET), could provide additional insights into microcirculatory health and should be considered in future research.

Thirdly, diagnostic uncertainty surrounding INOCA presents another significant limitation. INOCA can be challenging to diagnose accurately due to its heterogeneous presentation and the lack of specific diagnostic criteria. This uncertainty may lead to misclassification or underdiagnosis, potentially affecting the reliability of our findings regarding the incidence and risk factors of IRA‐INOCA in STEMI patients treated with DCB.

Lastly, the limitation stems from the retrospective nature of our study. Retrospective studies are inherently subject to biases such as recall bias and selection bias, which may impact the validity and generalizability of our results. Prospective studies with predefined protocols and rigorous data collection methods would be more robust in confirming our observations and exploring new hypotheses.

## Funding

This study was supported by the Nanjing Health Bureau Medical Science and Technology Development Foundation in China (No. YKK22101).

## Disclosure

The authors have nothing to report.

## Conflicts of Interest

The authors declare no conflicts of interest.

## Data Availability

The data that support the findings of this study are available from the corresponding author upon reasonable request.
